# Transesophageal Endoscopic Ultrasound With Bronchoscope‐Guided Fine‐Needle Aspiration for Tissue Diagnosis of Pleural Malignancy in a Patient With Respiratory Compromise

**DOI:** 10.1111/1759-7714.70416

**Published:** 2026-11-08

**Authors:** So‐Yun Kim, Chaeuk Chung, Dongil Park

**Affiliations:** ^1^ Division of Pulmonary, Allergy and Critical Care Medicine, Department of Internal Medicine Chungnam National University Hospital Daejeon Republic of Korea; ^2^ Department of Internal Medicine College of Medicine, Chungnam National University Daejeon Republic of Korea

**Keywords:** EUS‐B‐FNA, high‐flow nasal cannula, pleural malignancy, pleural metastasis, respiratory compromise, thoracic oncology

## Abstract

Histologic confirmation is essential in suspected pleural malignancy, particularly for subtype classification and molecular profiling. However, conventional diagnostic approaches, including thoracoscopic, percutaneous, and airway‐based bronchoscopic biopsy, may be difficult or high‐risk in patients with severe respiratory compromise or poor pulmonary reserve. Endoscopic ultrasound with bronchoscope‐guided fine‐needle aspiration (EUS‐B‐FNA) is not a standard diagnostic modality for pleural disease, and its role in sampling pleural lesions remains insufficiently characterized. A 71‐year‐old man with severe chronic obstructive pulmonary disease and a history of pneumothorax presented with progressive dyspnea. Chest computed tomography showed severe emphysema, left pleural effusion, and multifocal nodular pleural thickening suspicious for pleural malignancy. Pleural fluid cytology revealed malignant cells; however, tissue biopsy was required for definitive histologic classification and molecular testing. Despite pleural drainage, the patient remained dependent on high‐flow nasal cannula (HFNC) support because of persistent hypoxemia and hypercapnia. Conventional diagnostic procedures, including bronchoscopic biopsy, transthoracic needle biopsy, and thoracoscopic biopsy, were considered difficult or high‐risk because of severe bullous emphysema, marked dyspnea, respiratory failure, and the unavailability of medical thoracoscopy at our institution. As the pleural lesion was adjacent to the esophagus, EUS‐B‐FNA was performed under HFNC support. Histopathology confirmed poorly differentiated lung adenocarcinoma. The patient was ultimately diagnosed with stage IV lung adenocarcinoma with pleural metastasis. Transesophageal EUS‐B‐FNA may provide an alternative route for tissue acquisition in selected patients with suspected pleural malignancy when conventional biopsy is difficult or high‐risk and pleural lesions are anatomically accessible from the esophagus.

## Graphical abstract

A 71‐year‐old man with severe chronic obstructive pulmonary disease presented with suspected pleural malignancy and respiratory failure requiring high‐flow nasal cannula support. Conventional biopsy approaches were considered difficult or high‐risk because of severe emphysema, marked dyspnea, and poor pulmonary reserve. Transesophageal endoscopic ultrasound with bronchoscope‐guided fine‐needle aspiration (EUS‐B‐FNA) was performed to obtain tissue from a pleural lesion adjacent to the esophagus. Histopathologic examination confirmed poorly differentiated adenocarcinoma, establishing the diagnosis of Stage IVA lung adenocarcinoma with pleural metastasis and enabling molecular profiling. This case highlights the technical feasibility of EUS‐B‐FNA as an alternative route for tissue acquisition in carefully selected patients with anatomically accessible pleural lesions when conventional diagnostic approaches are challenging.

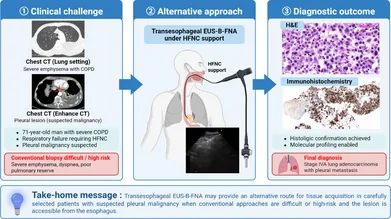

**Abbreviations**
CK7: cytokeratin 7
COPD: chronic obstructive pulmonary disease
CT: computed tomography
EUS‐B‐FNA: endoscopic ultrasound with bronchoscope‐guided fine‐needle aspiration
FDG PET/CT: fluorodeoxyglucose positron emission tomography/computed tomography
HFNC: high‐flow nasal cannula
MRI: magnetic resonance imaging
NSCLC: non‐small cell lung cancer
PCD: percutaneous drainage
TTF‐1: thyroid transcription factor‐1

## 1 Introduction

Obtaining adequate tissue is essential in patients with suspected pleural malignancy, as histopathologic confirmation is required not only for diagnosis but also for subtype classification and molecular testing. Conventional diagnostic approaches for pleural lesions include image‐guided percutaneous biopsy and thoracoscopy [1, 2, 3].

Among these approaches, thoracoscopy is widely recognized as an important diagnostic modality for pleural disease because it allows direct visualization of the pleura and targeted biopsy [1, 2]. However, although medical thoracoscopy is generally regarded as a minimally invasive procedure, it still requires entry into the pleural cavity, dedicated equipment, and procedural expertise, and may not be feasible in all patients [1, 2]. This may be particularly relevant in patients with severe respiratory compromise or poor pulmonary reserve.

Endoscopic ultrasound with bronchoscope‐guided fine‐needle aspiration (EUS‐B‐FNA) is an established technique for sampling mediastinal lesions and lung lesions adjacent to the esophagus, and its transesophageal route may provide an alternative means of tissue acquisition in selected patients with respiratory impairment [4, 5]. Although EUS‐B‐FNA is not a standard diagnostic modality for pleural disease, limited reports suggest that it may be a feasible alternative when a pleural lesion is located adjacent to the esophagus [6].

Here, we report a case of suspected pleural malignancy in a patient requiring HFNC support in whom conventional diagnostic approaches were considered difficult or high‐risk. EUS‐B‐FNA enabled transesophageal tissue acquisition from an esophagus‐adjacent pleural lesion and established the diagnosis needed for oncologic decision‐making.

## 2 Case Presentation

A 71‐year‐old man with a history of chronic obstructive pulmonary disease (COPD) and prior pneumothorax, which had been managed conservatively without chest tube drainage or surgery, presented to the emergency department with progressive dyspnea that had persisted for 3–4 days. He had a 70 pack‐year smoking history and had been poorly adherent to inhaler therapy despite being prescribed triple inhaler therapy with a long‐acting beta‐agonist, long‐acting muscarinic antagonist, and inhaled corticosteroid.

A chest radiograph obtained in the emergency department demonstrated a moderate to large amount of left‐sided pleural effusion (Figure 1A). Subsequent chest computed tomography (CT) revealed severe emphysema (Figure 1B,C), left pleural effusion, and multifocal nodular enhancing pleural thickening, raising suspicion for pleural seeding from an unknown primary malignancy (Figure 1D).

**FIGURE 1:**
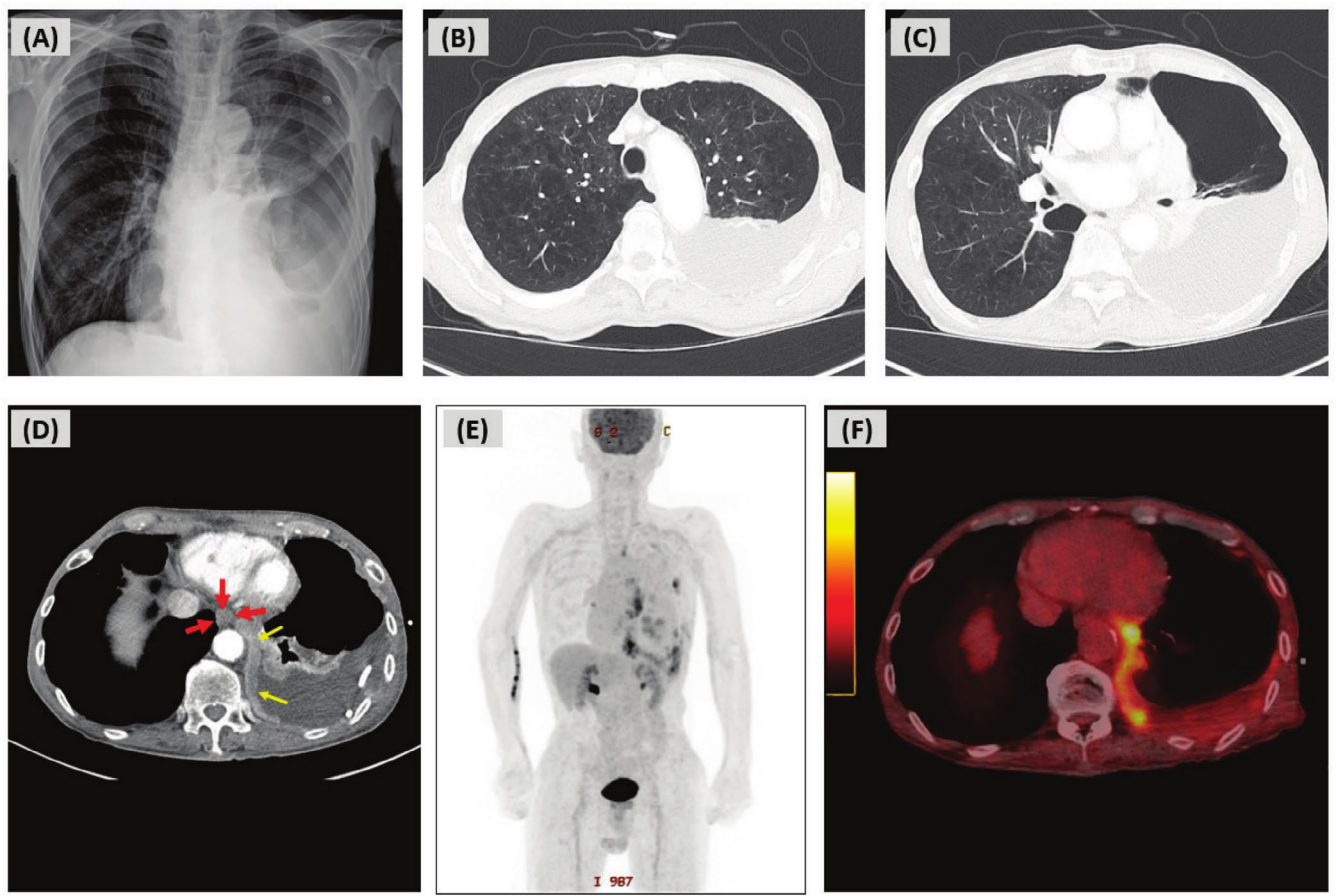
Radiologic findings of underlying severe emphysema and pleural‐based malignancy. (A) Chest radiograph showing a large left‐sided pleural effusion with associated lung collapse. Extensive lucent areas suggest underlying bullous emphysema. (B, C) Axial chest computed tomography (CT) images demonstrating severe emphysematous changes with diffuse low‐attenuation areas and bullae in both lungs, along with left pleural effusion. (D) Axial contrast‐enhanced chest CT image showing pleural‐based enhancing lesions along the left hemithorax (yellow arrows) in close proximity to the esophagus (red arrows). (E) Whole‐body fluorodeoxyglucose positron emission tomography/computed tomography (FDG PET/CT) maximum intensity projection (MIP) image demonstrating increased metabolic activity in the left thoracic cavity. (F) Axial fused FDG PET/CT image showing intense FDG uptake along the pleural‐based lesion.

A percutaneous drainage (PCD) catheter was inserted for the pleural effusion, and cytologic examination confirmed the presence of malignant cells.

To identify the primary tumor, whole‐body fluorodeoxyglucose positron emission tomography/computed tomography (FDG PET/CT) was performed, revealing increased FDG uptake along the left pleural surface, extending to the subpleural region of the left lower lobe (Figure 1E,F).

Although malignant cells were detected in the pleural fluid, thyroid transcription factor‐1 (TTF‐1) staining was negative. Given the need for histologic confirmation for subtype differentiation and molecular profiling, including next‐generation sequencing, tissue biopsy was considered essential.

Despite drainage of 2550 mL through the PCD catheter, the patient required high‐flow nasal cannula (HFNC) support (FiO_2_ 40%, flow rate 40 L/min) due to persistent hypoxemia and hypercapnia.

A 36‐mm lung lesion in the left lower lobe was suspected; however, the CT bronchus sign was unclear, and the surrounding emphysema was severe. Considering the patient's dyspnea and oxygen requirement, bronchoscopic biopsy or transthoracic needle biopsy was considered infeasible. Therefore, biopsy from the suspected pleural metastatic lesion was prioritized. However, medical thoracoscopy was not available at our institution. Given the anatomical proximity of the pleural lesion to the esophagus, EUS‐B‐FNA was selected as an alternative route for tissue acquisition (Figure 2A,B).

**FIGURE 2:**
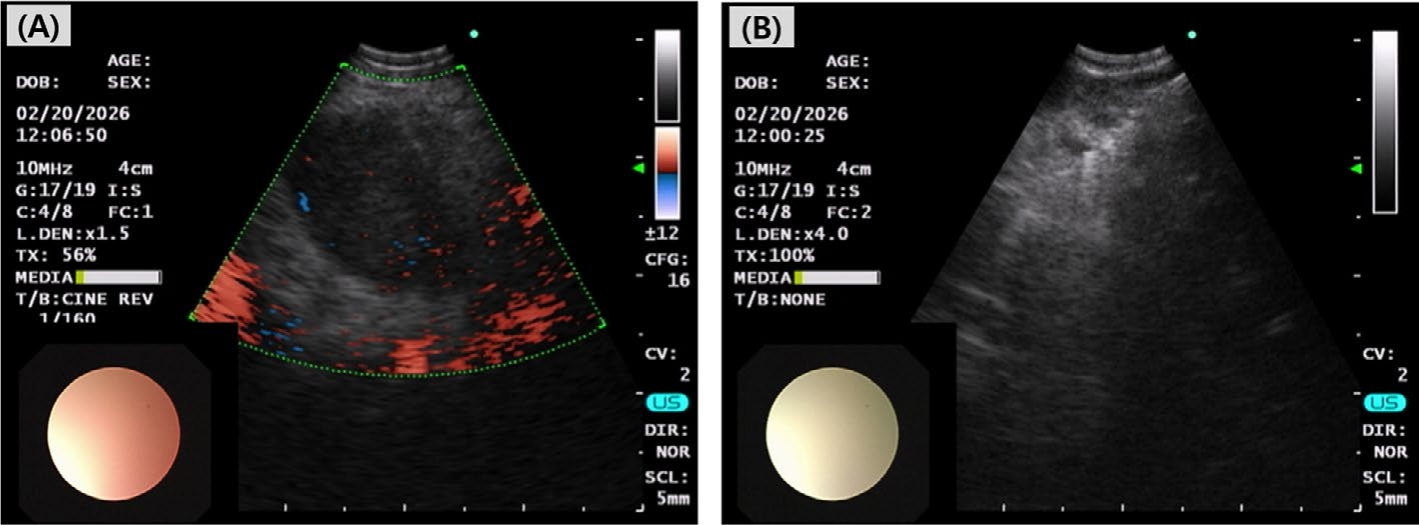
Endoscopic ultrasound with bronchoscope (EUS‐B) evaluation of the pleural mass. (A) EUS‐B with Doppler imaging demonstrates a heterogeneous hypoechoic lesion adjacent to the esophageal wall. (B) EUS‐B image shows an ill‐defined hypoechoic pleural mass visualized through the esophageal wall, which was subsequently targeted for EUS‐B‐guided fine‐needle aspiration.

EUS‐B‐FNA was performed in the bronchoscopy suite with the patient in the supine position using a linear‐array ultrasound bronchoscope (BF‐UC260FW) and an EU‐ME1 ultrasound processor (Olympus, Tokyo, Japan). Conscious sedation was achieved with intravenous midazolam (3 mg) and fentanyl (50 μg). During the procedure, HFNC support was maintained at a flow rate of 60 L/min with an FiO_2_ of 40%, with continuous electrocardiographic, pulse oximetry, and noninvasive blood pressure monitoring. The pleural lesion was identified transesophageally by delineating the thickened pleura and pleural effusion in relation to adjacent anatomical structures, including the descending aorta. A 22‐gauge EchoTip needle (Cook Medical, Bloomington, IN, USA) was initially attempted; however, its relative stiffness made it difficult to achieve an appropriate puncture angle. A 25‐gauge EchoTip needle was subsequently attempted, but satisfactory advancement into the target lesion could not be achieved. The needle was therefore changed to a 22‐gauge ViziShot 2 needle (Olympus), which allowed appropriate needle positioning and successful tissue acquisition while avoiding the adjacent aorta. Three separate needle passes were performed. For each pass, the needle was advanced into the target lesion, the stylet was removed, and multiple to‐and‐fro needle movements were subsequently performed under syringe suction before the needle was withdrawn. Rapid on‐site evaluation (ROSE) was not performed. The aspirated material was prepared for cytologic examination, including smears and cell‐block preparation, while the obtained tissue fragments were fixed in formalin and submitted for histopathologic examination. The procedure lasted 32 min. Peripheral oxygen saturation remained between 96% and 100% throughout the procedure and was 99% immediately after the procedure. No bleeding, clinically significant respiratory deterioration, or other immediate procedure‐related complications were observed.

Histopathologic examination of the EUS‐B‐FNA specimen revealed poorly differentiated adenocarcinoma (Figure 3A–C). Immunohistochemical staining showed positivity for MOC‐31 and cytokeratin 7 (CK7) (Figure 3D), while calretinin and p40 were negative (Figure 3E,F), supporting the diagnosis of non‐small cell lung cancer (NSCLC) with adenocarcinoma histology.

**FIGURE 3:**
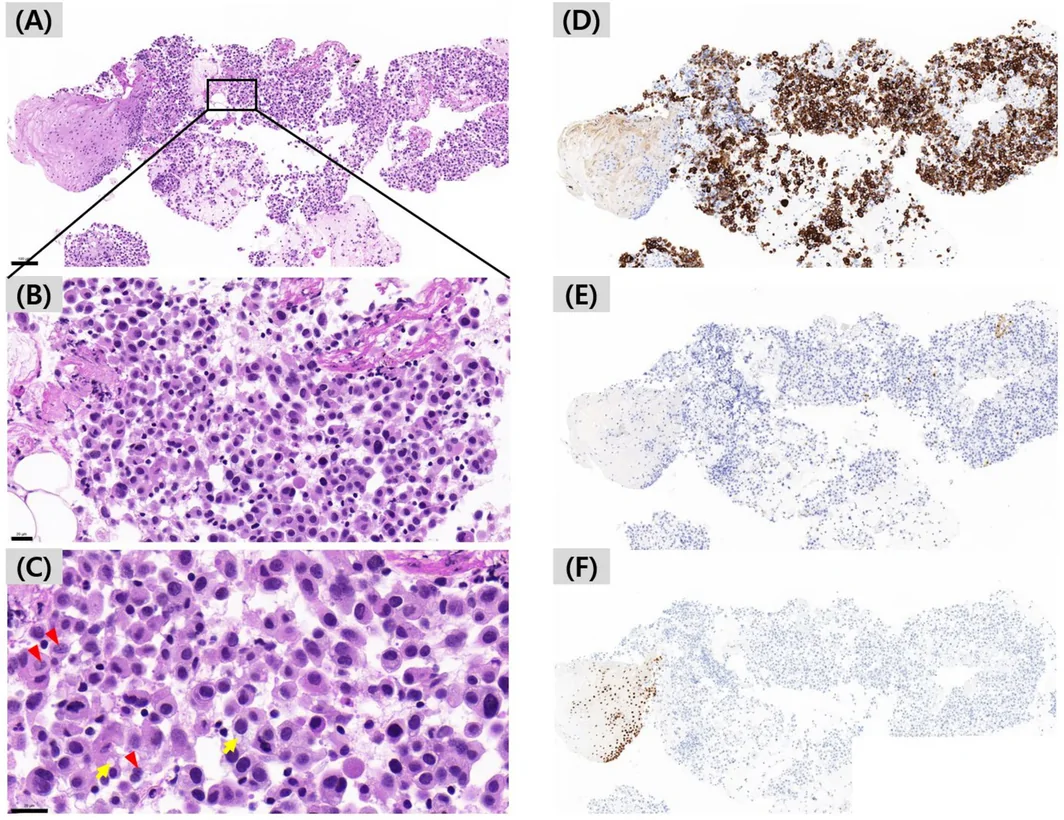
Histopathologic and immunohistochemical findings of the pleural tumor. (A) Low‐power view of the endoscopic ultrasound with bronchoscope‐guided fine‐needle aspiration specimen shows diffuse infiltration of atypical epithelial cells within fibrous stroma (hematoxylin and eosin [H&E] stain, ×10; scale bar = 100 μm). (B) High‐power view demonstrates sheets of atypical tumor cells with enlarged nuclei and prominent nucleoli (H&E stain, ×40; scale bar = 20 μm). (C) Higher magnification reveals tumor cells with nuclear enlargement and increased nuclear‐to‐cytoplasmic ratio (red arrowheads) and occasional intracytoplasmic vacuoles (yellow arrows), supporting poorly differentiated adenocarcinoma morphology (H&E stain, ×70; scale bar = 20 μm). (D) Immunohistochemistry shows diffuse positivity for cytokeratin (CK) (×10). (E) Calretinin staining is negative in tumor cells (×10). (F) p40 staining is also negative in tumor cells (×10).

No extrathoracic distant metastasis was detected on PET/CT or brain magnetic resonance imaging (MRI), while mediastinal nodal metastasis consistent with N3 disease was identified. The final clinical stage was cT2aN3M1a (Stage IVA) according to the 9th edition of the TNM classification for lung cancer, based on a 36‐mm primary tumor, contralateral mediastinal lymph node metastasis, and malignant pleural effusion. Molecular testing revealed no targetable mutations, and PD‐L1 expression was elevated. The patient received combination chemotherapy with carboplatin, pemetrexed, and pembrolizumab. However, the disease progressed rapidly, and the patient's respiratory status deteriorated despite treatment, ultimately leading to death.

## 3 Discussion

This case demonstrates the technical feasibility of transesophageal EUS‐B‐FNA as an alternative tissue acquisition approach for suspected pleural malignancy when the lesion is anatomically accessible from the esophagus [6], particularly in selected patients with severe respiratory compromise. In our patient, conventional diagnostic procedures were difficult to apply because of severe underlying emphysema, marked dyspnea, respiratory failure requiring HFNC, and the unavailability of medical thoracoscopy at our institution. Under these circumstances, EUS‐B‐FNA provided an alternative route for tissue acquisition and yielded sufficient material to establish the diagnosis.

Only a limited number of reports have described EUS‐B‐FNA for sampling pleural lesions (Table 1). In previously published cases, this approach was mainly applied to pleural lesions located adjacent to the esophagus or mediastinum, where transesophageal access was technically feasible. Several cases also shared a similar clinical context in which conventional diagnostic approaches, including thoracentesis, closed pleural biopsy, percutaneous biopsy, or endobronchial approaches, were nondiagnostic, technically difficult, or unsuccessful. Therefore, the existing literature suggests that EUS‐B‐FNA may be a feasible complementary diagnostic option in carefully selected patients with anatomically accessible pleural lesions.

**TABLE 1:** Published reports of EUS‐B‐FNA for sampling of pleural lesions.

Study	Age/sex	Underlying disease/comorbidities	Pleural lesion/location	Why conventional biopsy was difficult	Final diagnosis	Complications
Kassirer et al. [7]	60/M	20 pack‐year smoking history; type 2 diabetes mellitus; asbestos exposure	Pleural mass adjacent to the lower esophagus	Thoracentesis and closed pleural biopsy were nondiagnostic	Metastatic renal cell carcinoma	NR
Bibi et al. [6], Case 1	70/M	History of left‐sided MPM treated with pneumonectomy, partial pericardiectomy, and adjuvant chemotherapy 10 years earlier	Pleural lesion near the esophagus	Lesion location favorable for EUS‐B‐FNA	Malignant mesothelioma	NR
Bibi et al. [6], Case 2	75/F	History of adenoid cystic carcinoma of the left parotid gland, surgically resected	Pleural lesion near the esophagus	Lesion location favorable for EUS‐B‐FNA	Pleural metastasis from adenoid cystic carcinoma	NR
Tamburrini et al. [8], Case 1	67/M	Former smoker; hypertension; COPD; GERD; former metal worker	Subcarinal pleural thickening	Prior thoracentesis was negative for malignancy	Epithelioid malignant pleural mesothelioma	NR
Tamburrini et al. [8], Case 2	78/M	Former carpenter and smoker with COPD; prior right pleural decortication for empyema	Mediastinal pleural nodule	Pleural fluid analysis was inconclusive	Non‐Hodgkin B‐cell lymphoma	NR
Tamburrini et al. [8], Case 3	77/F	Non‐smoker; hypertension	Pleural thickening involving the mediastinum	NR	Epithelioid malignant pleural mesothelioma	NR
Juneja et al. [9]	62/M	EGFR‐mutated lung adenocarcinoma	Superior mediastinal pleural thickening posterior to the left subclavian artery	Percutaneous biopsy of a left upper lobe peripheral lesion was nondiagnostic	Pleural metastasis from lung adenocarcinoma	NR
Piro et al. [10]	49/F	No tobacco use or known asbestos exposure	Right lower paramediastinal pleural thickening extending into the mediastinum near the esophagus	Lesion location favorable for EUS‐B‐FNA	Epithelioid malignant pleural mesothelioma	NR
Eastburg et al. [11]	45/M	History of small cell carcinoma and adenocarcinoma of the lung	Right posteromedial pleural thickening above the right hemidiaphragm	Traditional biopsy methods, including an attempted endobronchial approach, were unsuccessful due to lesion location	Small cell carcinoma/adenocarcinoma involvement	NR
Present case	71/M	70 pack‐year smoking history; COPD; severe bullous emphysema; history of pneumothorax; respiratory failure requiring HFNC	Pleural mass/diffuse pleural thickening adjacent to the esophagus	Bronchoscopic biopsy, CT‐guided biopsy, and thoracoscopy were considered difficult or high‐risk because of severe emphysema and HFNC‐dependent respiratory failure	Adenocarcinoma	None

Abbreviations: COPD, chronic obstructive pulmonary disease; CT, computed tomography; EGFR, epidermal growth factor receptor; EUS‐B‐FNA, endoscopic ultrasound with bronchoscope‐guided fine‐needle aspiration; GERD, gastroesophageal reflux disease; HFNC, high‐flow nasal cannula; MPM, malignant pleural mesothelioma; NR, not reported.

In patients with suspected pleural metastasis or pleural‐based malignancy, thoracoscopy is considered an important diagnostic modality because it allows direct visualization of the pleura and acquisition of sufficient tissue samples [1, 2]. However, although medical thoracoscopy is generally regarded as a minimally invasive procedure, it still requires skin incision and entry into the pleural cavity, as well as dedicated equipment and procedural expertise [1, 2]. In contrast, EUS‐B‐FNA does not require an incision, can be performed through the esophagus rather than the airway, and does not require direct entry into the pleural cavity, providing a distinct route for tissue acquisition in anatomically suitable cases [4].

In patients with severe respiratory compromise, a potential rationale for the transesophageal route is that it allows tissue acquisition without airway instrumentation or partial airway obstruction by the bronchoscope. Previous case reports have demonstrated the feasibility of EUS‐B‐FNA in patients with markedly limited respiratory reserve. Ito et al. reported successful transesophageal bronchoscopic ultrasound‐guided needle aspiration in a patient with respiratory failure in whom conventional bronchoscopy was considered difficult [12], while Arimori et al. described the successful use of EUS‐B‐FNA in a patient with severe central airway stenosis after airway stenting [13]. These reports support the potential utility of the transesophageal route when airway‐based tissue acquisition is technically challenging or potentially hazardous. In our patient, severe bullous emphysema, marked dyspnea, and respiratory failure requiring HFNC complicated the use of conventional airway‐based and invasive diagnostic approaches, while the pleural lesion was anatomically accessible from the esophagus. Thus, EUS‐B‐FNA provided a technically feasible alternative route for tissue acquisition without transbronchial or transthoracic access. Nevertheless, the present report represents a single carefully selected case and does not establish that EUS‐B‐FNA is safer, better tolerated, or superior to conventional diagnostic approaches. Further studies are needed to determine its safety and clinical utility in patients with severe respiratory compromise.

Although EUS‐B‐FNA provides an alternative route for tissue acquisition, potential procedure‐related complications should be considered. Transesophageal needle puncture may be associated with bleeding, infection, aspiration, esophageal injury, mediastinitis, pneumothorax, and potential tumor seeding. In the present case, the target lesion and adjacent anatomical structures were carefully evaluated using real‐time ultrasonography and Doppler imaging, and the needle trajectory was selected to avoid major vascular structures, particularly the adjacent descending aorta. Needle advancement and sampling were performed under continuous ultrasound guidance. The patient was already receiving ceftriaxone before the procedure, and antibiotic therapy was continued after the procedure with consideration of the potential infectious risk associated with transesophageal puncture. Continuous cardiorespiratory monitoring was maintained throughout the procedure. No bleeding, clinically significant respiratory deterioration, infectious complications, or other immediate procedure‐related complications were observed. Nevertheless, the absence of complications in a single case does not establish the safety of this approach, and careful patient selection, anatomical assessment, and further evaluation of procedure‐related risks are warranted.

This approach also has clear limitations. EUS‐B‐FNA is feasible only when the lesion is sufficiently close to the esophagus and can be safely identified by ultrasound [5]. In addition, unlike thoracoscopy, it does not allow direct inspection of the pleura, and the amount of tissue obtained may sometimes be limited, which can restrict pathologic evaluation and molecular profiling [2, 3, 14].

## 4 Conclusion

Transesophageal EUS‐B‐FNA may represent an alternative route for tissue acquisition in selected patients with suspected pleural malignancy and poor pulmonary reserve or respiratory compromise, particularly when conventional bronchoscopic, percutaneous, or thoracoscopic approaches are difficult or potentially high‐risk and the lesion is anatomically accessible from the esophagus.

## Author Contributions

**So‐Yun Kim:** conceptualization, methodology, validation, investigation, resources, data curation, writing – original draft, writing – review and editing, visualization, supervision. **Chaeuk Chung:** validation, investigation, data curation, writing – review and editing, supervision. **Dongil Park:** conceptualization, methodology, validation, investigation, resources, data curation, writing – original draft, writing – review and editing, visualization, supervision.

## Funding

This work was supported by the National Research Foundation of Korea, No. RS‐2025‐24535457.

## Ethics Statement

The study was conducted according to the guidelines of the Declaration of Helsinki. Ethical review and approval were waived for this study due to its retrospective illustrative nature. It does not meet the national policy for the protection of human subjects' definition of research, which requires an investigation that contributes to generalizable knowledge about disease or condition.

## Consent

Written informed consent has been obtained from the patient to publish this paper.

## Conflicts of Interest

The authors declare no conflicts of interest.

## Data Availability

Data supporting the findings of this case report are contained within the article. Additional data are not publicly available because of patient privacy considerations.
